# C-Reactive Protein As a Mediator of Complement Activation and Inflammatory Signaling in Age-Related Macular Degeneration

**DOI:** 10.3389/fimmu.2018.00539

**Published:** 2018-03-15

**Authors:** Kathleen R. Chirco, Lawrence A. Potempa

**Affiliations:** ^1^Buck Institute for Research on Aging, Novato, CA, United States; ^2^Roosevelt University College of Pharmacy, Schaumburg, IL, United States

**Keywords:** C-reactive protein, age-related macular degeneration, inflammation, complement, complement factor H, membrane attack complex

## Abstract

Age-related macular degeneration (AMD) is a devastating neurodegenerative disease affecting millions worldwide. Complement activation, inflammation, and the loss of choroidal endothelial cells have been established as key factors in both normal aging and AMD; however, the exact mechanisms for these events have yet to be fully uncovered. Herein, we provide evidence that the prototypic acute phase reactant, C-reactive protein (CRP), contributes to AMD pathogenesis. We discuss serum CRP levels as a risk factor for disease, immunolocalization of distinct forms of CRP in the at-risk and diseased retina, and direct effects of CRP on ocular tissue. Furthermore, we discuss the complement system as it relates to AMD pathophysiology, provide a model for the role of CRP in this disease, and outline current therapies being developed and tested to treat AMD patients.

## Introduction

Age-related macular degeneration (AMD) is a progressive neurodegenerative disease affecting more than 8% of the global population (1), with roughly 11 million cases in the United States alone (2). Inflammation and complement activation are recognized as prominent events in the manifestation and progression of AMD, with C-reactive protein (CRP) as a potential mediator in these processes. Here, we review AMD pathophysiology, the complement system, and the current evidence supporting CRP as a contributor to complement activation and inflammation in the context of AMD. Finally, we will present a model for the role of CRP in this disease and provide insight into future therapies for AMD patients.

## AMD Pathophysiology

Early AMD is clinically identified by the presence of extracellular deposits, called drusen, and/or pigmentary changes within the macula, the central 6 mm of the retina. If the disease progresses, vision loss can occur either from atrophy of the retina (termed advanced dry AMD or atrophic AMD) or aberrant growth of vessels beneath the retina (termed wet AMD or neovascular AMD). The primary pathology in AMD includes dysfunction and/or loss of (1) the photoreceptor cells, which are the light-sensing cells of the retina, (2) the retinal pigment epithelium (RPE), which forms the outer blood–retinal barrier and consists of a single layer of epithelial cells that support photoreceptor cell function, and (3) the choriocapillaris, the capillary bed that lies just outside the RPE (and forms the innermost layer of the choroid) and provides oxygen and nutrients to both the RPE and photoreceptor cells.

While the pathologic changes that occur within the macula during AMD are incompletely understood, evidence supports complement-mediated changes and the loss of choroidal endothelial cells (CECs) as early events in both normal aging and disease (3, 4). CECs are believed to be lost early in disease progression, which may accompany or even precede dysfunction and loss of the RPE (4–9). Loss of these supporting cells eventually leads to loss of photoreceptor cells within the macula, causing significant and irreversible visual decline (10).

## The Complement System in AMD

Although AMD is a complex, multifactorial disease, many genetic factors have been associated with disease risk. To date, 19 genetic loci have been identified, including genes involved in regulating complement activity (11). The discovery of a genetic link between AMD and the complement system supports previous histological and proteomic data identifying complement proteins as constituents of drusen in postmortem eyes (12–14).

The complement system, which is important for eliminating pathogens, cellular debris, and dying host cells, consists of three distinct arms: the classical pathway, the alternative pathway, and the lectin pathway. After pathway-specific initiation, followed by a series of cleavage events, the pathways continue, similarly, through to cleavage of C5 and entrance into a common terminal pathway. The terminal pathway culminates in formation of the membrane attack complex (MAC), which promotes cell lysis. Similar to other arms of the immune system, the complement system must be carefully regulated, and various serum- and tissue-derived complement proteins aid in the regulation of one or more pathways. One of the major genetic risk loci for AMD includes the complement factor H (CFH) gene, which encodes an alternative pathway regulator and harbors multiple disease-associated variants (15). Importantly, the Y402H single nucleotide polymorphism (SNP) in *CFH* is a common variant that significantly increases AMD risk (15–18). The Y402H substitution occurs in the SCR7 region of the complement factor H (FH) protein, resulting in impaired protein binding to various substrates, including proteoglycans (19–22) and CRP (23).

Another component of the complement system, the MAC, has been linked to AMD through various histological studies. MAC deposition becomes increasingly elevated within the choriocapillaris with advancing age in human postmortem eyes, and this phenomenon is specific to the eye (24). MAC accumulation is further elevated in *CFH* Y402H high-risk and AMD maculae beyond what is observed in normal aging (3). Compromised binding of the FH Y402H protein to its extracellular matrix substrates, such as heparan sulfate proteoglycans, may increase complement activation in the human retina (21), and may be one cause for elevated MAC deposition in the macula in AMD (3, 4). The potential consequences of MAC accumulation in the choriocapillaris are twofold. First, MAC formation on CECs *in vitro* results in significant cell lysis. Second, the cells that survive complement attack adopt an angiogenic phenotype (25). While complement activation and MAC accumulation in the choriocapillaris in AMD are well-established events (26), the precise details for how and why these events occur remain to be fully elucidated.

## CRP in Complement Activation and Inflammation

C-reactive protein is an established component of drusen in human postmortem eyes (14, 27–29) and it plays a role in both complement activation and regulation (23, 30, 31), implicating CRP as an intriguing candidate for disease involvement.

Studies examining the role of CRP as a regulator of complement activation and other inflammatory response pathways have recently evolved to evaluate and distinguish the bioactivities of two distinct conformational forms of the protein. The widely appreciated serum-associated form is a hepatically synthesized pentameric discoid protein (pCRP) with high aqueous solubility and calcium-dependent phosphocholine (PC) binding affinity. pCRP can bind to PC groups exposed on disrupted plasma membranes, as occurs when lysophospatidyl choline is formed (32). Upon binding to disrupted cell membranes, as shown with activated platelets and apoptotic monocytes, pCRP undergoes rapid dissociation into monomeric form (mCRP) (33, 34) with distinctive solubility, antigenicity, tissue localization, binding ligands, and functions compared to pCRP (30, 35, 36). It is now known that mCRP rather than pCRP is an efficient activator of the classical complement pathway involving C1 (*via* binding to C1q), C4, C2, and C3 (37, 38). mCRP also acts as a regulator of the alternative complement pathway, *via* recruitment of FH to injured tissues. This binding to FH was shown to occur in a dose-dependent manner and at a site that does not interfere with mCRP binding to C1q (38). Importantly, the high-risk FH Y402H protein has reduced mCRP binding by up to 45% when compared to the FH Y402 protein (28, 39, 40).

In the context of atherosclerosis and coronary artery disease, mCRP has been shown to be strongly pro-inflammatory, differentiating monocytes toward a pro-inflammatory M1 phenotype (41), delaying neutrophil apoptosis, and stimulating leukocytes pro-inflammatory effector responses (42). In addition, Zouki and colleagues revealed a mCRP-mediated upregulation of CD11b/CD18 expression on the surface of human neutrophils, which results in enhanced adhesion of these cells to activated endothelium (43). Monomeric CRP can also activate human coronary artery endothelial cells *in vitro*, resulting in production of IL-8 and MCP-1 (44). Moreover, mCRP stimulates angiogenesis and promotes tube formation in brain microvasculature (45). While CRP has been widely studied outside the eye, the bioactivities of both forms of CRP are now beginning to be elucidated within ocular tissue in the context of AMD.

## Serum-Associated CRP as a Risk Factor in AMD

Seddon and colleagues began examining a potential link between CRP and AMD, by assessing CRP levels in the serum of AMD and control patients. They found CRP levels to be higher in individuals with intermediate and advanced AMD compared to controls (46), and they discovered a positive correlation between serum CRP levels and AMD progression (47). Furthermore, they found the lowest risk of AMD progression associated with CRP levels <0.5 mg/L, little change to AMD risk for CRP levels within the range of 0.5–10.0 mg/L, and the highest risk for AMD progression when CRP levels reached at least 10 mg/L (47). Finally, Seddon’s group examined the relationship between serum CRP levels, *CFH* genotype, and AMD risk (48), and they found CRP and *CFH* genotype to be independently associated with AMD risk. In fact, both the *CFH* variant and high CRP levels have an additive effect on AMD risk.

Interestingly, others have gone on to assess the relationship between serum CRP levels and AMD status in individuals harboring variants in the promoter region of the *CRP* gene, all of which either increase or decrease CRP levels in the serum. However, regardless of whether the SNP increased or decreased CRP levels, the authors found no direct association between any of the variants and AMD status (49–51). While none of these variants result in an amino acid change to the CRP protein and, therefore, do not affect biological function of the protein, these studies suggest that serum CRP levels alone may not be enough to alter AMD risk.

## CRP in the AMD Retina

The association between serum CRP levels and AMD risk may be important for defining disease biomarkers; however, the primary pathology of AMD occurs in the retina. Prior to the realization that CRP exists in more than one form (e.g., mCRP and pCRP), work done by Johnson and colleagues examined the presence of total CRP in the retina of postmortem eyes genotyped at the *CFH* locus (27). They showed that high-risk eyes (homozygous for the Y402H SNP) had more CRP immunoreactivity in the choroid compared to low-risk eyes (homozygous for Y402), especially in regions containing drusen-like deposits (27). A similar study aimed to determine differences in total CRP levels in the retina based on AMD status (52). CRP immunoreactivity differed based on disease status, with early and wet AMD eyes having more intense CRP immunolabeling compared to controls. However, in advanced dry AMD eyes with geographic atrophy (GA), CRP immunoreactivity in the non-atrophic area was similar to that of age-matched controls, with CRP levels significantly reduced within the atrophic lesions. CRP was primarily immunolabeling extending into Bruch’s membrane in early and wet AMD eyes (52). Interestingly, FH immunoreactivity was significantly reduced in the choroid in AMD eyes compared to controls, regardless of disease stage, suggesting an imbalance in CRP and FH levels, especially in early and wet AMD.

A recent study of human postmortem eyes examined CRP in the retina to determine which form of CRP is present in the tissue and to begin teasing out its precise role in AMD pathogenesis (29). Using monoclonal antibodies that clearly differentiate pCRP and mCRP antigens (53), the study found that mCRP is the primary form of CRP in the choroid, and it is predominantly localized to the choriocapillaris and Bruch’s membrane. Similar to previous work looking at total CRP (27), mCRP is more abundant in donor eyes with the high-risk *CFH* polymorphism compared to age-matched controls. Monomeric CRP also exhibits a direct effect on CECs *in vitro*, including increasing CEC migration and increasing monolayer permeability. Further-more, mCRP treatment of human RPE-choroid tissue *ex vivo* results in a significant upregulation of pro-inflammatory gene expression, including an increase in *ICAM1*, which has been associated with AMD previously (54). These data suggest a role for mCRP in promoting inflammation in the choroid, which may be especially true in individuals at risk for AMD development. This study revealed co-localization between mCRP and the MAC in the choriocapillaris of human postmortem eyes, providing evidence to support the hypothesis that mCRP increases complement activation in the choriocapillaris (29).

The work outlined above shows that mCRP primarily localizes to the choroid; however, other retinal cell types may be affected by the presence of this potent pro-inflammatory molecule within ocular tissue. For example, Molins et al. propose a role for mCRP in disruption of the outer blood–retinal barrier. They found that treatment with mCRP *in vitro* led to a disruption in tight junction integrity in ARPE-19 monolayers (55). Exposing ARPE-19 cells to mCRP also stimulates *IL8* and *CCL2* expression, two molecules that are important for leukocyte recruitment and blood–retinal barrier integrity (40, 56). Monomeric CRP binds necrotic RPE cells *in vitro* and enhances recruitment of FH to those cells by over 100% compared to FH recruitment without mCRP. In contrast, mCRP increases the recruitment of the Y402H variant of FH by just 50%. The mCRP-mediated recruitment of FH results in a 35% decrease in TNFα secretion from necrotic RPE cells, which is reduced to a 1% decrease for the high-risk FH molecule (28). These data indicate that without efficient recruitment of FH to RPE cells, the pro-inflammatory effects of mCRP may override the protective effects, leading to exacerbation of AMD pathology.

## Model for the Role of CRP in the Pathogenesis of AMD

**Figure 1:**
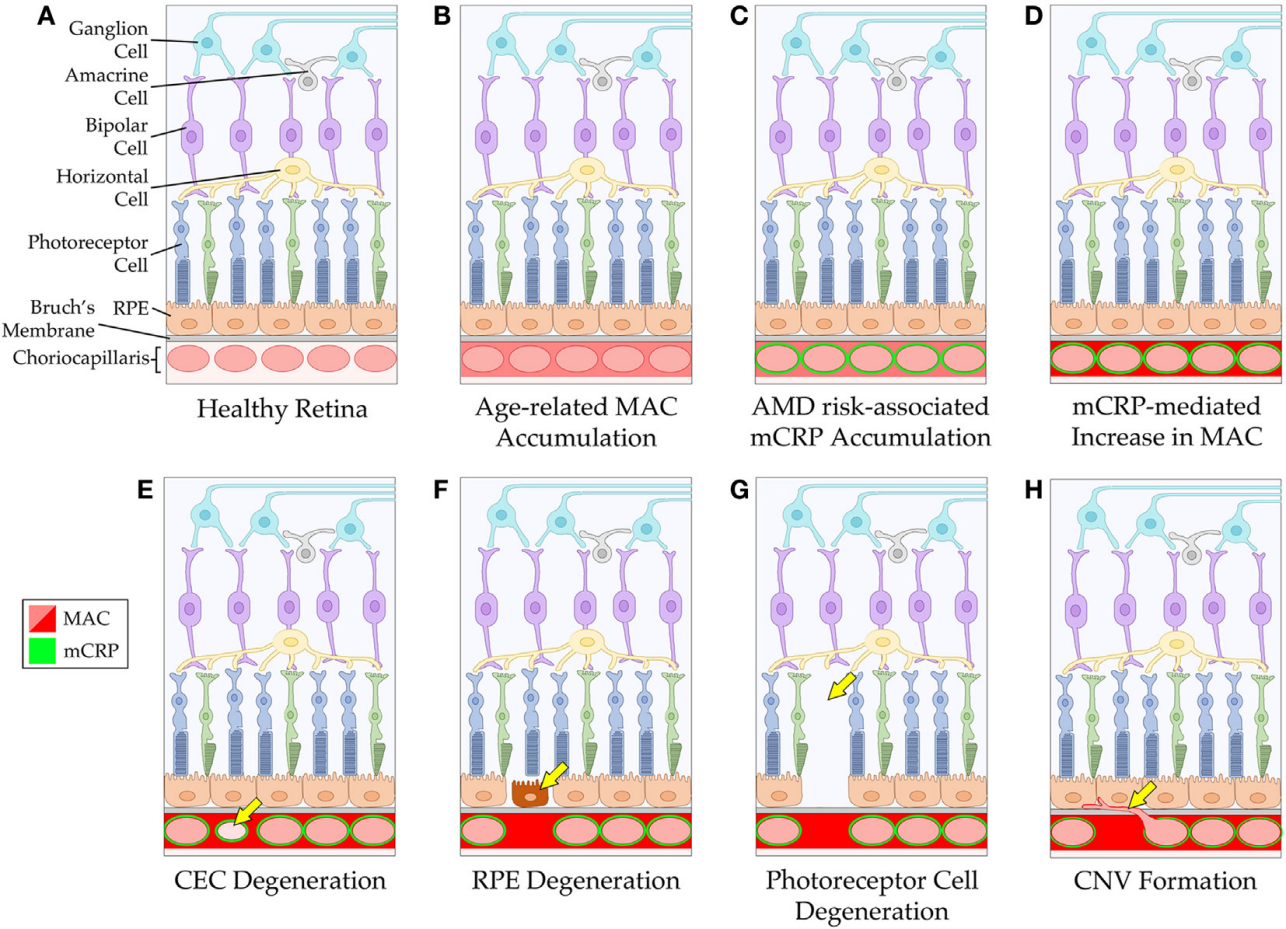
Schematic of mCRP-associated age-related macular degeneration (AMD) pathogenesis. The healthy retina and choriocapillaris is depicted in **(A)**. With advancing age, the membrane attack complex (MAC) accumulates around the vessels of the choriocapillaris **(B)**. In individuals with an increased genetic risk for AMD (*via* the *CFH* Y402H polymorphism), mCRP accumulates around the vessels of the choriocapillaris **(C)**, and this may lead to increased complement activation and subsequent elevation in MAC levels in the tissue **(D)**. The mCRP- and/or MAC-mediated changes to the tissue environment may result in CEC death and degeneration of the choriocapillaris **(E)**. Loss of the vessels of the choriocapillaris can cause dysfunction and degeneration of the RPE **(F)**, and eventually the photoreceptor cells **(G)**. Alternatively, loss of choriocapillaris vessels can lead to choroidal neovascularization formation **(H)**. RPE, retinal pigment epithelium; CEC, choroidal endothelial cell; CNV, choroidal neovascularization.

The amount of CRP that gets made during an acute phase response is up to 1,000-fold beyond baseline levels. While it is unlikely that all of the pCRP in serum gets into the tissue, it is possible that much of it does reach the target tissue and dissociate into monomers. Furthermore, local production of mCRP cannot be ruled out; however, more work is required to determine the precise source(s) of mCRP in the choriocapillaris. Regardless of the source, once mCRP reaches the choriocapillaris, it may presumably outnumber the regulatory FH molecules present, which could create an imbalance in complement regulation in favor of more complement activation. Furthermore, in the choriocapillaris, where complement injury is highest in AMD, FH is the primary regulator of the complement system. This is in contrast to the RPE, which has multiple complement regulators present and, therefore, may be better armed against complement-mediated injury (57, 58). In an individual who harbors the high-risk *CFH* polymorphism, their ability to control complement, at least the alternative pathway, is further limited due to altered binding capabilities of mutant FH to its tissue-associated ligands (e.g., glycosaminoglycans, such as heparan sulfate, within Bruch’s membrane). Since mCRP is shown to be present in these tissues, it is possible that mCRP may affect complement regulation resulting in the increased MAC deposition observed in the choriocapillaris with advanced age and disease. Together, these data propose a mechanism for CEC loss in AMD pathogenesis, *via* complement-mediated attack (Figure 1).

Evidence suggests that mCRP also plays a key role in promoting an inflammatory environment in AMD eyes. For example, mCRP increases *ICAM1* mRNA and protein levels in human postmortem RPE-choroid tissue *ex vivo* (29). ICAM-1, which is constitutively expressed in the choriocapillaris with highest levels in the macula (59), acts to promote leukocyte recruitment in the choroid (60) and elevated levels of ICAM-1 have been associated with AMD (54). Ultimately, combining the evidence for (1) its localization to the choriocapillaris in high-risk and AMD patients, (2) its role in complement system activation and its interaction with AMD-associated complement proteins, (3) its ability to directly promote CEC activation *in vitro* and *ex vivo*, and (4) its pro-inflammatory effects on RPE-choroid tissue, mCRP is a promising target for the treatment of AMD.

## Therapeutic Development to Target Inflammation and Complement Activation in AMD

Treatment options for AMD are currently limited, with the most effective therapies consisting of AREDS supplements (antioxidants plus zinc), which have been shown to reduce the risk of progression beyond early or intermediate AMD by about 25–30% over 5 years (61), and intravitreal anti-vascular endothelial growth factor injections, which can help ameliorate the symptoms of wet AMD (62). The absence of a more effective and universal therapy has been a driver for the continued pursuit of novel therapeutic targets. Based on the evidence for mCRP and complement activation as key players in AMD pathogenesis, future therapies may need to target both mCRP-mediated effects in addition to complement proteins to effectively treat the disease. Many clinical trials are already completed or underway to assess treatments that target inflammation or complement activation in individuals with AMD [Table 1; reviewed in Ref. (63, 64)]. For example, a handful of trials have aimed to target inflammation in participants with AMD using corticosteroids such as dexamethasone [NCT01162746] and fluocinolone acetonide (iluvien) [NCT00605423]. In addition to steroid therapies to reduce inflammation, various studies have taken aim at regulating complement system activation in AMD patients, including drugs targeting complement components C5, C3, and CFD.

**Table 1:** Completed and ongoing clinical trials to reduce inflammation and complement activation in age-related macular degeneration (AMD).

Therapy	Mechanism	Route	Target	Trial identifier
Ranibizumab + dexamethasone	Anti-VEGF + corticosteroid	Intravitreal injection	CNV	NCT00793923 NCT01162746
Dexamethasone	Corticosteroid	Intravitreal implant	CNV	NCT00511706
Fluocinolone acetonide (iluvien)	Corticosteroid	Intravitreal implant	AMD	NCT00605423
Eculizumab	Humanized monoclonal antibody targeting C5	IV infusion	GA	NCT00935883
ARC1905	Anti-C5 RNA aptamer	Intravitreal injection	GACNV	NCT00950638 NCT00709527
Zumira^®^	Anti-C5 aptamer	Intravitreal injection	GA	NCT02686658
LFG316	Humanized monocloncal antibody targeting C5	Intravitreal injection	GACNV	NCT01527500 NCT01535950
LFG316 + CLG561	Humanized monocloncal antibody targeting C5 + anti-properdin antibody	Intravitreal injection	GA	NCT02515942
POT-4/Compostatin	Inhibitor of C3 cleavage	Intravitreal injection	CNV	NCT00473928
Lampalizumab	Humanized monocloncal antibody targeting CFD	Intravitreal injection	GA	NCT02247479 NCT02247531
AAVCAGsCD59	sCD59 overexpression	Intravitreal injection	GA	NCT03144999

*GA, geographic atrophy; CNV, choroidal neovascularization*.

Despite the promise these ongoing trials provide, many hurdles still exist in the therapeutic regulation of inflammation and complement in AMD patients, including delivery method, dose, and disease stage at time of treatment. The use of gene therapy to treat AMD may help resolve these current issues and provide a promising option for future treatments. Early studies to examine gene therapy-mediated treatments are already underway in mice [e.g., Cr2-fH fusion protein (65) and FH overexpression (66)] and in human clinical trials (AAVCAGsCD59; NCT03144999). Additional gene therapy options, such as CRISPR/Cas9-mediated gene editing, may be useful to correct high-risk variants in AMD patients, including the *CFH* Y402H polymorphism. Since advanced AMD pathology includes loss of multiple cell types within the macula, cell replacement therapy, possibly in combination with other gene editing or drug therapies, could provide the greatest promise for improved visual acuity in AMD patients. As we continue to advance our understanding of the key mediators in AMD pathogenesis, we continue to move closer to the development of life-changing treatments for millions of individuals.

## Author Contributions

KC and LP wrote and edited the manuscript.

## Conflict of Interest Statement

The authors declare that the research was conducted in the absence of any commercial or financial relationships that could be construed as a potential conflict of interest.
